# New Trends, Advantages and Disadvantages in Anticoagulation and Coating Methods Used in Extracorporeal Life Support Devices

**DOI:** 10.3390/membranes11080617

**Published:** 2021-08-12

**Authors:** Anne Willers, Jutta Arens, Silvia Mariani, Helena Pels, Jos G. Maessen, Tilman M. Hackeng, Roberto Lorusso, Justyna Swol

**Affiliations:** 1 ECLS Centre, Cardio-Thoracic Surgery, and Cardiology Department, Heart & Vascular Centre, Maastricht University Medical Centre (MUMC), P. Debyelaan 25, 6229 HX Maastricht, The Netherlands; s.mariani1985@gmail.com (S.M.); j.g.maessen@mumc.nl (J.G.M.); roberto.lorusso@mumc.nl (R.L.); 2 Cardiovascular Research Institute Maastricht (CARIM), Maastricht University, Universiteitssingel 50, 6229 ER Maastricht, The Netherlands; t.hackeng@maastrichtuniversity.nl; 3 Engineering Organ Support Technologies Group, Department of Biomechanical Engineering, Faculty of Engineering Technology, University of Twente, P.O. Box 217, 7500 AE Enschede, The Netherlands; j.arens@utwente.nl (J.A.); h.n.pels@student.utwente.nl (H.P.); 4 Department of Biochemistry, Faculty of Health, Medicine and Life, Maastricht University, P.O. Box 616, 6200 MD Maastricht, The Netherlands; 5 Department of Respiratory Medicine, Allergology and Sleep Medicine, Paracelsus Medical University, Ernst-Nathan Str. 1, 90419 Nuremberg, Germany

**Keywords:** extracorporeal life support, extracorporeal membrane oxygenation, anticoagulation, circuit modifications, coating methods

## Abstract

The use of extracorporeal life support (ECLS) devices has significantly increased in the last decades. Despite medical and technological advancements, a main challenge in the ECLS field remains the complex interaction between the human body, blood, and artificial materials. Indeed, blood exposure to artificial surfaces generates an unbalanced activation of the coagulation cascade, leading to hemorrhagic and thrombotic events. Over time, several anticoagulation and coatings methods have been introduced to address this problem. This narrative review summarizes trends, advantages, and disadvantages of anticoagulation and coating methods used in the ECLS field. Evidence was collected through a PubMed search and reference scanning. A group of experts was convened to openly discuss the retrieved references. Clinical practice in ECLS is still based on the large use of unfractionated heparin and, as an alternative in case of contraindications, nafamostat mesilate, bivalirudin, and argatroban. Other anticoagulation methods are under investigation, but none is about to enter the clinical routine. From an engineering point of view, material modifications have focused on commercially available biomimetic and biopassive surfaces and on the development of endothelialized surfaces. Biocompatible and bio-hybrid materials not requiring combined systemic anticoagulation should be the future goal, but intense efforts are still required to fulfill this purpose.

## 1. Introduction

Extracorporeal life support (ECLS) devices are used for cardiac or/and pulmonary support as a bridge to recovery, bridge to surgery or treatment, to decision, or to transplant in the presence of cardio-circulatory or respiratory refractory compromise. Overall, hospital survival of adult patients undergoing ECLS for respiratory support is reported to be 69% while survival in cardio-circulatory support is 59% [1]. 

**Figure 1:**
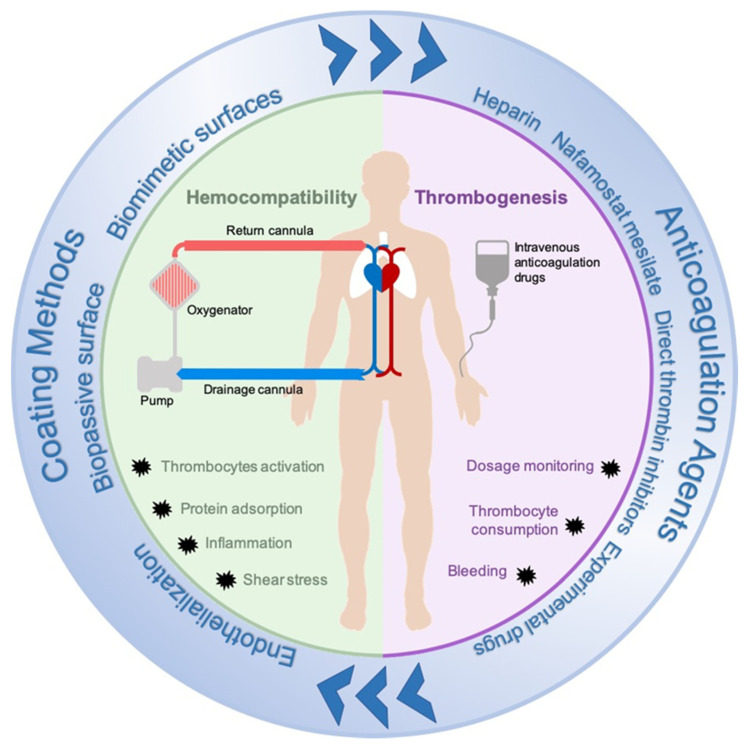
Visual summary of the interaction between hemocompatibility and thrombogenesis during extracorporeal life support and the adopted strategies to control it through anticoagulation agents and coating methods.

The effects of ECLS assistance, however, are not consistently positive. Compared to cardio-pulmonary bypass (CPB), ECLS devices provide support for several days or weeks. Consequently, blood is exposed to the artificial tubing and membrane surfaces for a long time, leading to activation of the patient’s inflammatory response and coagulation [2]. Prolonged ECLS duration may increase the risk of clot formation, which can result in severe complications (e.g., oxygenator failure, thrombosis, or emboli) and are associated with a decreased survival to discharge. Indeed, clotting inside the circuit or vessels thrombosis may occur, such as in the case of oxygenator failure reported in 9.1% and 6.6% of respiratory and cardiac adult patients, respectively [3,4]. Thus, anticoagulation is necessary to prevent these adverse events. Bleeding events are also frequently reported, and they are twice as common as thrombotic events [3]. Therefore, improvement of ECLS clinical results is necessary bonded to the reduction of thrombotic and hemorrhagic adverse events. Based on the complex interaction between the patient’s homeostasis and the ECLS circuit, these two players are the main targets to be addressed to prevent thrombo-embolic problems. Indeed, in the last decades, efforts have been done to develop new anticoagulant medications able to reduce embolic events while preventing bleedings in the patient´s body. Similarly, ECLS components and materials have been modified to improve their hemocompatibility and reduce the effects of blood-material contact. The interaction between hemocompatibility and thrombogenesis during extracorporeal life support and the adopted strategies to control it through anticoagulation agents and coating methods are summarized in Figure 1.

Despite significant improvements, clinical evidence highlights the persistent need for further research on hemocompatibility and anticoagulation agents in ECLS. This narrative review provides a state-of-the-art overview of currently available anticoagulation agents, the most recent circuit hemocompatibility improvements, and their expected future developments. 

## 2. Materials and Methods

To provide a broad presentation of the anticoagulation strategies and available coatings for ECLS, a search of PubMed/Medline was performed from inception to March 2021. Terms used for the search included ‘Extracorporeal Life Support’, ‘Anticoagulation’, ‘Heparin’, ‘Unfractionated heparin’, ‘Thrombin inhibitors’, ‘Hirudin’, ‘Nafamostat Mesilate’, ‘Factor Xa inhibitors’, ‘Factor IIa inhibitor’, ‘Coatings’, ‘Circuit surfaces’, and ‘Endothelialization’.

We included randomized clinical trials, controlled before-and-after studies, prospective and retrospective cohort studies, cross-sectional studies and case-control studies, reviews, and animal studies. Conference abstracts, books or grey literature, articles not written in English were excluded. Articles reporting on anticoagulation methods in patients supported with ECLS and research papers on coatings of ECLS components were retrieved. References were scanned for further information.

Based on the original study design, a group of experts was convened to openly discuss the references retrieved from the literature. The final evidence was summarized as a narrative review. 

## 3. Results

### 3.1. Anticoagulation Agents

To minimize the risk of thrombosis or clotting in the circuit, and subsequently the failure of the ECLS system, patients receive systemic anticoagulation. An optimal anticoagulation agent should be easy to administer and monitor and have a moderate risk for bleeding complications while maintaining the anti-thrombotic effects. Moreover, it should have an antidote or short half-life to ensure possible counteraction or fast extinguishing effect. Currently, multiple anticoagulant drugs are available, but each of them has specific advantages and disadvantages, implying the fact that the perfect agent still needs to be found (Table 1).

Currently used anticoagulation agents can be divided into three groups: heparin group, nafamostat group, and direct thrombin inhibitors. Other anticoagulants have been described in experimental models or case-reports and include recombinant forms of hirudin, oral anti-coagulants and experimental factor XIIa antibodies.

#### 3.1.1. Clinically Used Anticoagulation Agents

##### Unfractionated Heparin

The most commonly used anticoagulation during ECLS is unfractionated heparin (UFH). It has an inhibitory effect by binding the enzyme inhibitor antithrombin and increasing its inhibitory potential toward coagulation enzymes factor Xa and thrombin [5,6]. UFH is administered continuously and usually titrated based on activated clotting time (ACT), anti-factor Xa activity levels, or activated partial thromboplastin time (aPTT) [5]. Though, these measurements do not always correlate correctly with the heparin dose and effect, leading to some uncertainty in the monitoring of patients’ anticoagulation status [7]. Anti-Xa does correlate superiorly on heparin concentrations compared to ACT and aPTT, on the other hand, it does not represent the overall hemostatic state of the patient [8]. Thromboelastography (TEG) and thromboelastometry (ROTEM) have been studied in ECLS populations, where ROTEM showed moderate correlation with standard coagulation test and [9] ROTEM has been found to be a good indicator of anticoagulation status in pediatric patients undergoing ECLS as well [10]. Furthermore, UFH might stimulate the development of antibodies against heparin-platelet factor 4 complexes, which induce heparin-thrombocytopenia and thrombosis (HITT) [11]. The incidence of HITT varies between 0.36% [12] and 3.1% [13], and 50% of ECLS patients diagnosed with HITT develop clinically significant thrombotic events if no alternative anticoagulant is given [12]. While circulating UFH is surely related to HITT, it is unclear if heparin-coated circuits may induce HITT [14]. 

Regardless, in the case of HITT, alternative anticoagulants should be administered, and all sources of heparin should be removed, including heparin-coated components [12,15]. In addition, protamine sulfate can be administered to reverse the effects of UFH. To summarize, UFH is still the most used anticoagulation agent used in ECLS patients but its monitoring uncertainty and the risk of HITT prompt exploring of new anticoagulant agents [16]. 

##### Nafamostat Mesilate

A possible alternative for UFH is nafamostat mesilate (NM). NM is a synthetic serine protease inhibitor, often used as an anticoagulant for patients with a high bleeding risk on hemodialysis. It inhibits thrombin, factor Xa, and XIIa, the kallikrein-kinin system, complement system, and lipopolysaccharide-induced nitric oxide production. There is no antidote available, but NM has a short half-life of 8–10 min [17]. 

A study comparing NM to UFH in dogs on ECLS revealed decreased hemoglobin levels after 1 hour of ECLS in all animals. However, the NM group experienced no cannulation site bleeding as opposed to the UFH group. Thrombo-elastography and aPTT results were comparable between groups, but pro-inflammatory cytokine levels were lower with NM [18]. A retrospective study of patients on ECLS showed a longer duration of oxygenators, less transfusion of red blood cells, fresh frozen plasma, and cryoprecipitate when NM was used as an anticoagulation agent compared to UFH. In addition, the rate of bleeding, thrombosis, and mortality was higher in the heparin group [19]. Similarly, Han et al. observed more bleedings with UFH, but 3 cases of intracerebral hemorrhage with NM. Survival was higher in the NM group (38.2% vs. 13.6%) and heparin was found to be the only independent predictor of bleeding complications [20]. Conflicting results were presented in another retrospective study based on propensity-matched data. In this case, bleeding events occurred more in the NM group, probably because of the lack of an antidote for NM [21]. In conclusion, evidence on NM is still controversial and it is mainly used as an alternative anticoagulation agent, especially in patients with a high bleeding risk on hemodialysis. 

##### Direct Thrombin Inhibitors

Direct thrombin inhibitors are known alternatives for heparin in HITT patients. These agents bind directly to thrombin and inhibit the actions of thrombin, including feed back-activation of factors V, VIII, and XI, and conversion of fibrinogen to fibrin, and the stimulation of platelets [22]. 

**Bivalirudin**, a synthetic hirudin, is a direct thrombin inhibitor peptide often used as anticoagulation in HITT patients or patients with heparin resistance [6]. There is no antidote available, however the half-time of bivalirudin is 25 min and the onset of action is within 4 min [23]. It is mostly cleared by the kidneys and dosages should be adjusted in renal dysfunction [22,24]. It can be monitored by aPTT but also with ROTEM [25]. Bivalirudin has been used as an off-label anticoagulation therapy in ECLS with no significant increased risk of bleeding or thrombosis [24]. In post-cardiotomy ECLS patients, bivalirudin-based anticoagulation, compared to conventional heparin, has been associated with less bleeding and transfusion rates [26]. Similar outcomes were found in a mixed ECLS adult cohort, where bivalirudin showed less bleeding complications and a lower rate of thrombosis compared to heparin. In the same study, heparin was associated with higher aPTT variations compared to bivalirudin [27]. Indeed, it has been demonstrated that time within the therapeutic range is better with bivalirudin, especially in high-intensity anticoagulation protocols [28]. On the other hand, other studies failed to show the significant superiority of bivalirudin in terms of mortality and adverse events. For example, Kaseer et al. were not able to demonstrate any differences in 30-day and in-hospital mortality, major bleedings, renal and hepatic impairment, and thrombotic events between heparin and bivalirudin [29]. Again, bivalirudin showed more consistency than heparin in ACT and aPTT levels without higher risk for bleeding in patients with normal hepatic function [29,30]. However, dose adjustment is required in patients with hepatic impairment due to possible false and unpredictable aPTT prolongation and changes [31]. Different dosages of bivalirudin have been reported in studies with ACT and aPTT as monitoring tools to test the effect of medication [30]. Indeed, the optimal bivalirudin dosage still needs to be defined.

**Argatroban** is a small molecule direct thrombin inhibitor and can also be an alternative for UFH in patients with a contraindication for UFH and renal failure. Differently from bivalirudin, argatroban binds to the active site of thrombin (univalent), whereas bivalirudin binds to the active site and an additional exosite-1 on thrombin (bivalent) [22]. The onset of action is within 30 min and the half-life of this agent is around 45 min, with no antidote available [24]. Argatroban is eliminated by hepatic metabolism, and liver dysfunction requires dosage change [22,32,33]. No randomized controlled trials are available on argatroban, and its clinical use is justified based on case series and case reports [24]. A preclinical study showed lower fibrinolytic levels and higher platelet count in animals treated with argatroban compared to heparin and supported with CPB, using circuit components with or without heparin coating [34]. Another study tested three sham ECLS circuits with blood priming and demonstrated that thrombin formation was lower in the argatroban anticoagulated circuits compared to heparin, despite a less prolonged aPTT [35]. Even in ARDS patients requiring ECLS, argatroban administration was found feasible and safe, and comparable to heparin. Outcomes of bleeding complications, requiring transfusion, thrombotic complications, and replacement of ECLS components did not differ between heparin or argatroban anticoagulated patients [36]. The use of argatroban has been reported in patients simultaneously receiving continuous renal replacement therapy (CRRT) and veno-venous (V-V) ECLS. In these patients, a dosage of 2 µg/kg/min resulted in bleeding complications, and lowering the dose to 0.2 µg/kg/min showed promising effects [33]. The use of argatroban is associated with higher aPTT values and requires more frequent measurements to titrate the drug to an optimal therapeutic level [37].

As for bivalirudin, a standard dosage for argatroban is still difficult to be defined. 

#### 3.1.2. Anticoagulation under Investigation

##### Low Molecular Weight Heparin

Low molecular weight heparin (LMWH) has been described as anticoagulation during ECLS with promising results in clinical trials, even if its use is uncommon. The standard test for monitoring LMWH is an anti-Xa essay [38]. Thromboelastography is an assay to measure the stages of clot development and has also been described as a monitoring assay for LMWH. However, it has not been proven superior to anti-Xa assays. ROTEM does not fully detect the effects of LMWH [38,39]. Since LMWH selectively targets factor Xa through antithrombin, it has more predictable pharmacokinetics and therefore does not need routine monitoring [40]. The risk for HITT is also lower with LMWH [7]. Krueger et al. reported a rate of 18% relevant bleeding complications in 61 patients undergoing V-V ECLS support for 7 days with only LMWH as anticoagulation. In 4 (6.5%) patients severe thrombotic events occurred, but all after more than 5 days of ECLS [41]. In lung transplantation patients, similar outcomes were found. Of 102 patients with perioperative ECLS during lung transplant 80 patients received LMWH, and the remaining 22 received UFH as anticoagulation. No significant differences in bleeding complications were found between both groups, but thromboembolic events occurred more often in the UFH group [40]. LMWH seems promising, but it is difficult to predict the ending of its effect in the case of need and it cannot be considered as an alternative to UFH in the case of HITT due to the potential remaining risk of HITT antibody formation [42]. 

##### Recombinant Forms of Hirudin

Hirudin has been reported as a possible alternative for UFH. It is a naturally occurring anticoagulant in the salivary glands of leeches, and different recombinant (and synthetic) forms are available as anticoagulants but none of them is paired to an antidote.

**Lepirudin** is a recombinant form of hirudin. It is a bivalent direct thrombin inhibitor, binding to the catalytic site and exosite-1 of thrombin. It is approved by the Food and Drug Administration (FDA) as an alternative drug for heparin in the occurrence of HITT. The half-life of lepirudin is 1–2 h and administration by bolus can increase aPTT to a maximum within 10 min. Due to the renal elimination route, dosages must be adjusted in acute kidney injury [43]. This agent has been used in patients undergoing ECLS with contra-indications for UFH. The literature reports two pediatric cases of lepirudin use in patients diagnosed with HITT and suffering from biventricular heart failure requiring ECLS [44]. Another two cases reported on lepirudin use in adults with similar conditions [45,46]. In both cases, aPTT and ACT were used to titrate dosages, and, in one case, a lower dose was required based on acute kidney injury. In all described patients, no bleedings or thromboses occurred. Since 2013, lepirudin is no longer available on the market [24]. 

**Desirudin** is another recombinant-DNA form of hirudin with an irreversible inhibition action to thrombin. It has been proven to be more effective than UFH or LMWH in reducing the risk of deep venous thrombosis [47] and to have a similar effect compared to argatroban in the treatment of HITT [48]. However, there are no case reports or case series discussing the use of desirudin during ECLS. 

Due to these agents’ exogenous protein character, an immune reaction can be triggered and cause anaphylaxis [43].

##### Direct Oral Anticoagulants

Direct factor Xa inhibitors, such as rivaroxaban, apixaban, edoxaban (factor Xa inhibitors), and direct thrombin inhibitors such as dabigatran are direct oral anticoagulants (DOACs) or non-vitamin K antagonist oral anticoagulants (NOACs) used for secondary prophylaxes in atrial fibrillation and treatment of deep venous thrombosis (DVT) and venous thromboembolism (VTE). One case-report report addressed the uneventful use of rivaroxaban for 10 days in a COVID patient on V-V ECLS with suspected HITT, with no other intravenous anticoagulation alternatives. In this case, anti-Xa assays were used to monitor the rivaroxaban levels [49]. So far, no further evidence for the use of direct factor Xa inhibitors in ECLS as anticoagulation is available [50]. 

### 3.2. Circuit Modifications: Coating Methods

The complex interaction between inflammation and coagulation significantly affects a patients’ safety, but it has also important consequences on the ECLS devices as well, especially in terms of durability. Despite the routine patient’s systemic anticoagulation, deposition of blood proteins onto the artificial ECLS surfaces may still occur, leading to inefficient membrane functioning, insufficient gas transfer, and finally, device failure [51]. This is a major limitation for the long-term use of ECLS systems and a major obstacle toward the development of totally implantable durable devices [52,53]. The main limiting factors are related to platelet and coagulation activation leading to clot formation within the system, and protein adsorption which gradually impairs gas exchange in the oxygenator [52]. For these reasons, research efforts are aiming to improve hemocompatibility of foreign surfaces, optimize gas and blood flows, miniaturize ECLS systems, and decrease the imbalance of coagulation and inflammation [52]. 

From an engineering point of view, the new ECLS circuits should aim to mimic the physiologic conditions in order to avoid hemolysis and reduce the shear stress and/or the stasis zones [54,55,56,57]. The artificial surface area of the ECLS systems should be minimized by simplifying the circuit, reducing shear stress and stasis, while maintaining or increasing usability [58]. On the other hand, the ultimate goal is to mimic healthy endothelial tissue in circuits´ surfaces such as oxygenators´ membranes and housing parts, pumps, cannula, and tubing to eliminate both the systemic inflammatory and the coagulation pathway responses. 

Normally, anticoagulant regulation of procoagulant processes is regulated by the endothelium which is absent at the artificial surfaces of the ECLS circuit. The artificial surfaces not only activate platelets and factor XII, but also adsorb plasma proteins like fibrinogen, immunoglobulins, hemoglobin, fibronectin, and van Willebrand factor, in varying amounts depending on the material, but especially on hydrophobic surfaces [59]. This protein adhesion is thought to be the initiating factor of the procoagulant response [60]. As a consequence, to improve the hemocompatibility of these artificial ECLS surfaces, a replication of the anti-thrombotic and anti-inflammatory properties of the endothelium would be ideal. According to Ontaneda and Annich, surface modifications addressing this goal can be classified into three major groups [61]: bioactive surfaces (also called biomimetic surfaces); biopassive surfaces; and endothelialization of blood-contacting surfaces.

An overview of the commercially available hemocompatibility improving coatings for extracorporeal circulation systems is available in Table 2.

#### 3.2.1. Bioactive Surfaces

**Heparin-coated** systems for ECLS were developed to reduce the hemorrhagic risk by lowering the systemic heparinization [62,63,64,65]. The first heparin coating to become commercially available was developed by the company Carmeda in 1983 [66,67]. From that time on, several new coatings with different bonding techniques have been developed and became available in the market. The local release of heparin can minimize the negative effects of foreign materials coming in contact with blood [68]. In an early study, Videm et al. found that heparin coatings have the ability to reduce complement activation by 45% [69]. Wendel and Ziemer analyzed several studies and assumed that oxygenators coated with heparin can reduce the following effects in comparison to uncoated devices: activation of contact activation of coagulation, complement system activation, alteration of granulocytes, inflammation, and pulmonary complications, activation of platelets, disturbance of homeostasis, loss of blood, and cerebral damage [70]. However, the utility of heparin-coated materials has been questioned. Covalently- and ionic-bonded heparin coating on oxygenators reduced some effects of the inflammatory response, thrombi formation, but other complications remained the same when compared to uncoated oxygenators [60]. In general, these studies need to be interpreted with some caution as most were performed either in 6 h in vitro tests or in short-term use in CPB. Thus, their relevance for long-term ECLS is limited, but no evident contraindications are reported so far [71].

**Nitric Oxide (NO)** is also known as an endothelium-derived relaxing factor and is released by endothelial cells to induce vasodilatation. NO activates an increase in cyclic guanosine monophosphate (GMP) in platelets and vascular smooth muscle cells [61]. Indeed, coatings with NO-catalytic bioactivity can inhibit collagen-induced platelet activation and adhesion, proliferation, and migration of arterial smooth muscle cells through the cGMP signaling pathways. Studies showed good anti-thrombogenic properties in extracorporeal circuits [61,72]. Moreover, stents implanted in rabbits with this coating showed improved endothelial mimetic microenvironment, stronger recovery to the endothelium, and had less restenosis and thrombosis after 4 weeks [73]. A significant reduction in platelet consumption and activation was also observed in animal studies. The latest generation of NO coating is characterized by a lipophilic NO donor complex embedded into plasticized PVC to prevent uncontrolled NO release in the circulatory system. This technology showed not only platelet inhibition but also less fibrinogen consumption. The main disadvantage with NO is the fact that its storage cannot exceed 4 weeks. This can be a problem in long-term ECLS runs [61,72]. So far, NO-coatings have not been used commercially. However, NO was clinically used as a fraction of the sweep gas (20 ppm) of the oxygenator in 31 pediatric ECLS runs in order to use its anti-thrombotic properties by diffusion through the gas exchanger membrane [74].

To further improve hemocompatibility, a novel covalent **C1-esterase inhibitor (C1-INH)** coating has been introduced by Gering et al. [53]. Besides complement inhibition, C1-INH also prevents factor XII (a) activation, an early event of contact phase activation at the crossroads of coagulation and inflammation [53]. This coating is still under development and thus not commercially available.

#### 3.2.2. Biopassive Surfaces

**Albumin** has been used as coating material since 1980 and it is often indicated in case of contraindications from heparin [75]. Albumin coating is used as a base layer with a hydrophilic surface, which reduces the biological response to hydrophobic surfaces [23]. Albumin lacks binding sequences for platelets, leukocytes, and coagulation enzymes and therefore slows down the platelet activation when used as a coating. Nevertheless, albumin coatings do not last long due to displacement by procoagulant proteins [75]. Some manufacturers use albumin as part of a multi-layer, bioactive coating in alternating layers with heparin (Table 2: Bioline and X.ellence coatings).

**Phosphorylcholine (PC)** is anti-thrombogenic, protein resistant, antibacterial, and has anti-fouling properties [67]. Coatings with phosphorylcholine (PC) have been developed as an alternative to heparin-bound systems. PC is a hydrophilic polar headgroup of phospholipids. It contains a negatively charged phosphate bonded to a positively charged choline. Phospholipids containing PC are non-thrombogenic. PC coatings in extracorporeal circuits have been found to induce plateau formation of thromboxane B2 and thromboglobulin and even reduce thrombin formation [76]. However, other studies did not find PC favorable over heparin-coated circuits [61]. A study by Thiara et al. compared heparin-albumin coating with PC coating in elective cardiac surgery patients. The PC group showed significantly higher lactate dehydrogenase, thus hemolysis, but this was allocated to the fact that the group had significantly longer aortic clamping time and CPB duration. Further, hemoglobin, platelet counts, numbers of leukocytes and cytokines, levels of complement activation, and endothelial shedding molecule syndecan-1 were not significantly different between the two coating groups [77].

**Poly(2-methoxy-ethyl-acrylate) (PMEA)** is a blood-compatible polymer composed of a hydrophobic polyethylene chain and a mild hydrophilic tail. This combined hydrophobic and hydrophilic polymer allows the polymer to adhere to the hydrophobic site to different materials and create a hydrophilic surface for the blood to contact with the other side. Proteins and platelets will not denature or adhere to the hydrophilic surface [59]. Animal studies involving CPB revealed suppression of the complement system activation [61]. Compared to non-coated systems in patients undergoing coronary artery bypass grafting, PMEA coating was superior in reduction of platelet adhesion, aggregation, and protein adsorption [78]. However, other studies found a higher risk of postoperative leukopenia and systemic inflammatory response syndrome (SIRS) without a decrease in platelet aggregation [79]. Finally, there is no consensus on whether or not PMEA is superior to heparin-bound systems.

**Polyethylene oxide (PEO)**, commercially used in combination with negatively charged sulphonate groups and sulphate, is used as a biopassive coating, which has been proposed as an alternative to the heparin-loaded coatings. In an ex vivo study with human blood (n = 40), Teliguia et al. found no differences in coagulation activation (factor IIa, prothrombin fragment 1 + 2 were assessed) when compared to a heparin coating. All groups demonstrated similar adhesion scores following ultrastructural oxygenator assessment by scanning electron microscopy and no difference in the pressure gradients of the oxygenators was observed [80].

**Poly(MPC-co-BMA-co-TSMA) (PMBT)**, a zwitterionic copolymer, is also a polymer with both positive and negative charged components [81]. PMBT coating was shown to be stable on polypropylene hollow fiber membranes, tested by Wang et al. by elution with ethanol and washing and sterilizing solutions of peracidin. In the same study in animal models, almost no change in fibrinogen and platelets in the blood after blood circulation through PMBT copolymer circuits was observed. In the uncoated circuits, fibrinogen and platelets were significantly reduced due to absorption and consumption. Thrombus formation was significantly lower in the PMBT circuits. PMBT’s influence on gas exchange was not tested in the study [82]. The mimetic surface seems promising and might be applicable in artificial lung systems, however, it is not commercially available yet.

In an in vitro study by Preston et al., different coatings were tested in ECLS circuits with bovine blood. Coatings were tested regarding the adsorption of morphine and fentanyl. Safeline^®^ coating—a synthetic albumin (Maquet), Softline^®^ coating—a heparin free polymer (Maquet), Bioline^®^ coating—recombinant albumin and heparin (Maquet), Xcoating^®^—poly2methoxylacetylate (Terumo), Carmeda^®^ coating—covalently bonded heparin (Metronic), and Trillium^®^—covalently bonded heparin (Metronic) were compared to one another. All circuit coatings were associated with the loss of drugs. The Carmeda^®^ and Xcoating^®^ had significantly more morphine adsorption than Safeline^®^, Softline^®^, Bioline^®^ and Trillium^®^. Fentanyl was adsorbed more in Safeline^®^, Softline^®^, Bioline^®^, and Trillium^®^ compared to Carmeda^®^ and Xcoating^®^, but was not statistically significant [83].

#### 3.2.3. Endothelialization

Surface endothelialization is a technique where an endothelial layer is created onto circuit surface areas by seeding cells onto the surface to achieve complete hemocompatibility between blood and materials. Creating a surface with endothelial cells would achieve higher hemocompatibility than replicating specific thrombo-regulatory aspects of the endothelium. Few studies have investigated the feasibility of establishing an endothelial monolayer on the gas exchange ECLS membranes [51], although it is known that endothelial cells do not adhere easily to hydrophobic surfaces [75]. To provide an endothelial monolayer, the base of the material must enable endothelial attachment and bonding while preserving the viability of the endothelial cells. Heparin/albumin-coated PMP membrane fibers were found to be a good base for a viable and confluent endothelial monolayer of endothelial cells. Moreover, the heparin/albumin coating avoids thrombogenic events in areas not covered with cells [84]. Pflaum et al. demonstrated the effectiveness of a stable titanium dioxide (TiO_2_) coating achieved by pulsed vacuum cathodic arc plasma deposition (PVCAPD) technique on hydrophobic poly(4-methyl-1-pentene (PMP) membranes, with a functional monolayer of endothelial cells as a result. Although the use of the TiO_2_ coating resulted in a reduction in the oxygen transfer rate (OTR) of the membrane by 22%, it successfully mediated EC attachment. The endothelial layer was resistant to shear stress and able to repair itself when monolayer disruption appeared. [51]. A study experimented with endothelial cell seeding from cells derived from juvenile sheep carotid arteries and searched for the best protein coating for endothelial cell attachment. Seeding endothelial cells to uncoated oxygenator membranes was ineffective, and using gelatin, fibrinogen, and collagen IV did not enhance the cell seeding process. Cornellissen et al. considered fibronectin to be a good base for cell attachment on flat sheet membranes, however, they did not perform gas exchange performance tests [85]. However, current research on how to establish a single layer of endothelial tissue on the gas exchange of ECLS equipment is not advanced [23]. In addition, the shelf life of an endothelialized oxygenator can, under hypothermic conditions, be stretched up to two weeks [86] compared to the shelf life of an otherwise coated oxygenator being typically 2 years. This would result in complex resource planning and management for both manufacturers and ECLS centers. The use of immune-silenced cells might at least help in quicker response times as production for a particular patient would not depend on the availability of autologous cells. Indeed, Wiegmann et al. showed that the rejection of allogeneic endothelial cells could be prevented by silencing HLA-class I expression [87]. However, many questions in relation to costs, timely production, quality assurance, and approval of endothelialized oxygenators remain open, leaving a wide field of potential research.

## 4. Future Perspective and Conclusions

Since the first successful ECLS application, technological and medical progress has led to a wide application of ECLS devices with improved patient outcomes. As the evolution process of ECLS systems continues, the application of this support is likely to increase in the future, based also on the growing population suffering from acute and chronic heart and lung failure. To further improve the ECLS circuits, the aim is to find the materials that are comparable to the human body, require no or limited anticoagulation (thereby limiting bleeding-related complications), and do not initiate a thrombogenic and inflammatory response without compromising the oxygenation. It is thus mandatory to prompt the research field toward the development of better anticoagulant molecules and improved ECLS components. A combination of stable ECLS anti-adsorbant and anti-coagulant coatings with (low dose) systemic anticoagulant and antiplatelet therapy might be an optimal first line of defense against ECLS-induced thrombotic and bleeding complications. 

In parallel, new ECLS bio-hybrid materials are being developed to prevent the initiation of the thrombogenic and inflammatory response triggered by the blood–surface interaction, without compromising the gas exchange process. With the onset of the endothelialization technique, creating complete biocompatible materials seems achievable. For example, 3D stem cell printing is a technique on the rise even though the limited life span of the stem cells and long-term engraftment remain a major difficulty [88]. 

Overcoming these problems could lead to further use of life support systems, without risk for systemic inflammatory reactions and with less need for anticoagulation. Finally, this will make possible the development of totally implantable lung and heart devices and long-term ECLS without interferences to the hemostasis of the body. 

## Figures and Tables

**Table 1:** Overview of the different anticoagulation agents described for clinical and experimental use in extracorporeal life support.

	Anticoagulation Agent	Inhibition Site	Monitoring	Half-Time	Antidote	Advantages	Disadvantages
**Clinically Used Anticoagualtion Agents**	Unfractionated Heparin	Factor Xa and thrombin inhibition	Anti-factor Xa, ACT, aPTT	1–3 h	Protamine-sulfate	Saturable clearance mechanism and renal clearance, widely used most experience	Risk of HITT, variable effects on APTT, no linear effect
	Nafamostat mesilate	Serine protease inhibitor	ACT, aPTT	8–10 min	No antidote	Short half time anti-inflammatory effect	No large prospective trials available, short half time, higher costs than UFH
	Bivalirudin	Direct thrombin inhibitor	ACT, aPPT, PTT	25 min	No antidote	Renal clearance no risk for HITT easy titration	May interfere with APTT, less effective inhibition in areas of stasis
	Argatroban	Direct thrombin inhibitor	ACT, aPTT	45–50 min	No antidote	Hepatic clearance No risk for HITT Good dose response	Can interfere with INR, lesser coagulation inhibition in areas of stasis
**Anticoagulant Agents Under Investigation**	Low-molecular-weight-heparin	Factor IIa and Xa inhibition	Anti-factor Xa, aPTT	3–6 h	Protamine-sulfate	lower risk of HITT partially effective	AntiXa levels, accumulation in renal impairment
	Lepirudin	Direct thrombin inhibitor	ACT, aPTT, ECT	1–2 h	No antidote	Renal clearance No risk for HITT	Limited evidence in ECLS, risk for anaphylaxis, no longer available
	Rivaroxaban	Direct-Xa inhibitor	Anti-factor Xa	5–9 h	Andexanet alfa	Rapid onset of action, few drug interactions	No clear laboratory monitoring available, only oral administration possible

Abbreviations: ACT: Activated Clotting Time, aPTT: activated Partial Thromboplastin clotting Time, HITT: Heparin Induced Thrombocytopenia and Thrombosis.

**Table 2:** Overview of the commercially used coatings in extracorporeal life support circuit components.

	Main Coating Compount(s)	Commercial Name of Coating	Company
** Bioactive **	Heparin	Cortiva Bioactive surface	Medtronic
	Heparin	Rheoparin	Xenios/Fresenius
	Albumin + Heparin	Bioline	Maquet/Getinge
	Albumin + Heparin	X.ellence	Xenios/Fresenius
** Biopassive **	Albumin	Rheopak	Chalice Medical
	Albumin	Recombinant Albumin Coating	Hemovent
	Albumin	Safeline (discontinued)	Maquet/Getinge
	Albumin	X.eed	Xenios/Fresenius
	Phosphorylcholine	PC phosphorylcholine	Eurosets
	Phosphorylcholine	PH.I.S.I.O Coating	Liva Nova
	poly(2-methoxyethylacrylate) (PMEA)	Xcoating	Terumo
	Sulphate and sulphonate groups and polyethylene oxide (PEO)	Balance Biosurface	Medtronic
	Sulphonate groups, polyethylene oxide (PEO) and heparin	Trillium Biosurface	Medtronic
	Amphyphilic polymer	Softline	Maquet/Getinge

## Data Availability

Not applicable.
